# Herbivorous dinosaur jaw disparity and its relationship to extrinsic evolutionary drivers

**DOI:** 10.1017/pab.2016.31

**Published:** 2016-12-15

**Authors:** Jamie A. MacLaren, Philip S. L. Anderson, Paul M. Barrett, Emily J. Rayfield

**Affiliations:** 1 Department of Biology, Universiteit Antwerpen, Campus Drie Eiken, Universiteitsplein, Wilrijk, Antwerp, 2610, Belgium; 2 Department of Animal Biology, University of Illinois at Urbana–Champaign, 515 Morrill Hall, 505 S. Goodwin Ave., Urbana, Illinois 61801, U.S.A.; 3 Department of Earth Sciences, Natural History Museum, London, Cromwell Road, London, SW7 5BD, U.K.; 4 * School of Earth Sciences, University of Bristol, 24 Tyndall Avenue, Bristol, BS8 1TQ, U.K.

## Abstract

Morphological responses of nonmammalian herbivores to external ecological drivers have not been quantified over extended timescales. Herbivorous nonavian dinosaurs are an ideal group to test for such responses, because they dominated terrestrial ecosystems for more than 155 Myr and included the largest herbivores that ever existed. The radiation of dinosaurs was punctuated by several ecologically important events, including extinctions at the Triassic/Jurassic (Tr/J) and Jurassic/Cretaceous (J/K) boundaries, the decline of cycadophytes, and the origin of angiosperms, all of which may have had profound consequences for herbivore communities. Here we present the first analysis of morphological and biomechanical disparity for sauropodomorph and ornithischian dinosaurs in order to investigate patterns of jaw shape and function through time. We find that morphological and biomechanical mandibular disparity are decoupled: mandibular shape disparity follows taxonomic diversity, with a steady increase through the Mesozoic. By contrast, biomechanical disparity builds to a peak in the Late Jurassic that corresponds to increased functional variation among sauropods. The reduction in biomechanical disparity following this peak coincides with the J/K extinction, the associated loss of sauropod and stegosaur diversity, and the decline of cycadophytes. We find no specific correspondence between biomechanical disparity and the proliferation of angiosperms. Continual ecological and functional replacement of pre-existing taxa accounts for disparity patterns through much of the Cretaceous, with the exception of several unique groups, such as psittacosaurids that are never replaced in their biomechanical or morphological profiles.

## Introduction

Sauropodomorph and ornithischian dinosaurs were the foremost herbivorous terrestrial vertebrates of the Mesozoic Era in terms of species richness, abundance, and functional diversity (Weishampel and Norman 1989; Sereno 1999; Weishampel et al. 2004; Barrett 2014). Both groups survived two extinction events—the end-Triassic mass extinction (Tr/J) and a smaller extinction at the Jurassic/Cretaceous boundary (J/K)—and persisted through several episodes of floral turnover, including the decline of cycadophytes and the proliferation of angiosperms (Sereno 1997; Barrett and Willis 2001; Lloyd et al. 2008; Butler et al. 2009b). However, relatively few studies have attempted to quantify the responses of nonavian dinosaurs to these extrinsic environmental drivers.

A number of studies have investigated the ecological and evolutionary responses of dinosaurs to the Tr/J mass extinction in terms of diversity analyses, but only a handful of studies have quantified morphological disparity (Brusatte et al. 2008a,b) or the evolution of other traits across this interval (Irmis 2011; Sookias et al. 2012). These studies found that dinosaur morphospace occupation was not greatly affected by the Tr/J extinction (Brusatte et al. 2008a,b): dinosaurian disparity remained essentially unchanged across the Tr/J boundary, whereas crurotarsans became almost completely extinct (Brusatte et al. 2008a). With respect to dinosaurs the J/K extinction has been studied in terms of diversity analyses (e.g., Upchurch and Barrett 2005; Barrett et al. 2009; Butler et al. 2010, 2011; Upchurch et al. 2011), and the potential ecological consequences of this event have been discussed qualitatively in terms of changes to dinosaur browsing regimes and community composition (Bakker 1978; Barrett and Willis 2001; Barrett and Upchurch 2005). Possible associations between paleobotanical turnovers and dinosaur evolution have been proposed (e.g., Bakker 1978; Weishampel and Norman 1989; Tiffney 1992; Mustoe 2007), with the suggestion that changes in the prevalent mode of dinosaur herbivory (e.g., high-browsing vs. low browsing; extensive oral processing vs. lack of oral processing) were reciprocally related to changes in the taxonomic and ecological composition of contemporary plant communities. In particular, it has been suggested that a decline in sauropodomorph and stegosaur abundance and diversity might be associated with a decline in cycadophyte diversity during the Early Cretaceous and that the ecological radiation of angiosperms during the same period may have been fostered by a coincident taxonomic radiation of low-browsing ornithischian dinosaurs with complex jaw mechanisms (e.g., Bakker 1978; Weishampel and Norman 1989; Tiffney 1992; Mustoe 2007). Hypotheses regarding dinosaur–plant coevolution have been more recently tested quantitatively and qualitatively using spatiotemporal comparisons between the dinosaur and paleobotanical records (Barrett and Willis 2001; Butler et al. 2009a,b, 2010). These diversity-based spatiotemporal studies found no definitive evidence for the coradiation of any Mesozoic plant and dinosaur group, although some temporal correlations were suggestive of possible interactions. Physiological limits on some of these coevolutionary hypotheses have also been proposed on the basis of the possible nutritional value of potential food plants (e.g., Hummel et al. 2008; Gee 2011).

Disparity analyses quantify morphological diversity within a group of organisms, rather than merely documenting taxonomic richness (Wills et al. 1994; Ciampaglio et al. 2009). Unlike species-richness estimates, disparity analyses can be robust to sampling biases and document the variation in morphology and potential function within taxonomic groups (Wills et al. 1994). Assessments of morphological disparity using either anatomical measurements or cladistic characters have been conducted on various extinct vertebrate groups, including dinosaurs (Brusatte et al. 2008a,b, 2012; Young and Larvan 2010; Butler et al. 2011; Foth and Rauhut 2013; Button et al. 2014). By contrast, a new method for assessing the diversity of biomechanical profiles, multivariate biomechanical disparity (Anderson 2009; Anderson et al. 2011, 2013; Stubbs et al. 2013), has not been widely applied. Biomechanical disparity offers a novel means to quantify variation in biomechanically relevant traits and to infer their potential ecological significance: for example, biomechanical traits might include mechanical advantage (the ratio of muscle moment arms indicating the efficiency of force transfer during biting), polar moment of inertia (a proxy for flexural stiffness), and mandibular articulation offset (dictating simultaneous occlusion of the entire tooth row, or scissor-like occlusion) (Anderson 2009; Anderson et al. 2011, 2013; Stubbs et al. 2013). Other studies have explored disparity of individual biomechanical traits such as mechanical advantage (Sakamoto 2010; Brusatte et al. 2012), average maximum stress, or a metric of skull strength (Foth and Rauhut 2013). Continuous measurements can be projected into multivariate “biomechanical morphospace.” Previous work in this area has used two-dimensional (2D) views of mandibular elements to investigate the appearance and diversity of biomechanical profiles during the radiation of Paleozoic fishes (Anderson 2009; Anderson et al. 2011), the water-to-land transition in tetrapods (Anderson et al. 2013), the Mesozoic diversification of crocodylomorphs (Stubbs et al. 2013), and niche partitioning in sauropod dinosaurs (Button et al. 2014).

Despite previous work, the functional responses to these potential evolutionary drivers, and hence how the organism interacted with its environment and potential drivers of selection, have not been quantified. Without this information we lack a complete picture of how dinosaur communities and clades interacted with and exploited Mesozoic environments over time. In addressing these questions, assessing the morphological variation evident from the fossil record may not be sufficient, as we do not know whether morphology and morphological diversity are reliable predictors of function and functional diversity. Therefore, in order to assess the relationship between jaw shape, function, and extrinsic evolutionary drivers, we provide the first quantitative assessment of the morphological and biomechanical disparity of an individual functional unit (the lower jaw) in herbivorous nonavian dinosaurs through time. This approach complements previous attempts to examine these questions though spatiotemporal comparisons of species-richness patterns and provides the only rigorous biomechanically and functionally based analysis of these issues attempted to date. We hypothesize that ornithischians and sauropodomorphs will show distinct morphologies and biomechanical profiles (i.e., in both the shape and mechanical capabilities of the jaw). We also hypothesize that the shift in plant community structure after the J/K boundary will trigger a corresponding shift in dinosaurian jaw biomechanical profiles, due to the differing physiognomies, digestibility, and mechanical properties of the varied potential food plant clades that were ecologically important at different times throughout the Mesozoic (Bakker 1978; Weishampel 1984; Niklas 1992; Hummel et al. 2008; Gee 2011). We use a geometric morphometric landmark analysis to compare dinosaur mandibular shape variability to variation in mandibular biomechanical profiles. We then compare these data with the timing of several extrinsic events (tetrapod extinctions, changes in floral communities) that have been proposed to influence dinosaur evolutionary history, in order to determine whether coincident patterns are present.

## Materials and Methods

Data for 2D landmark and biomechanical trait analyses were compiled from 167 sauropodomorph and ornithischian dinosaur taxa (see Supplementary Information, Appendix 6). Herbivorous nonavian theropods were excluded from this data set, as complete mandibular material for these animals is rare. A mandibular biomechanical profile represents a good proxy for characterizing the feeding system, as the mandible is primarily adapted for feeding, whereas the cranium has multiple functional roles, some of which are unrelated to feeding, such as housing the brain and sensory organs (Hylander et al. 1991; Hylander and Johnson 1997).

### 

#### Morphology

The archosaur mandible is a primarily planar structure, although its morphology does differ between groups, with varying degrees of inturning and bowing, particularly with respect to its symphyseal region (Romer 1956). However, to include as many taxa as possible, in order to account for the greatest amount of biomechanical and mandibular and dental shape variation, we selected a standard lateral view of the mandible as the basis for this study. The 2D landmarks were applied to homologous and analogous points on lateral images of dinosaur jaws using tpsDig II software (Rohlf 2004; Zelditch et al. 2012). Six fixed landmarks were described, identifying biologically and operationally homologous points on both sauropodomorph and ornithischian jaws (see Supplementary Fig.1). The overall morphology of each jaw was described by a series of sliding semilandmarks (sLM). Six sLM curves, each bracketed by two of the fixed landmarks, were used to define the shape of the jaw. In total, 88 landmarks (both fixed and sliding) were described. sLMs were slid using the Chord-d^2^ technique to minimize Procrustes distances rather than bending energy (Rohlf 2008); this was performed in tpsRelw. Described curves were appended to landmarks in tpsUtil (Rohlf 2004); appended landmarks were then superimposed using generalized least-squares (Procrustes) methods in tpsRelw (Rohlf 2008). Procrustes superimposition aligned jaws, eliminating scale, location, and rotational differences between specimens (Rohlf 2004). Consensus models, partial warps, and relative warps were then calculated using tpsRelw software. Relative warp scores were subjected to principal components analysis (PCA) to produce shape-based morphospace plots.

#### Biomechanics

Eighteen continuous biomechanical characters or traits were quantified, many of which have important functional consequences in extant organisms (Table 1). Full details of the biomechanical characters are described in the Supplementary Material. Biomechanical trait measurements were standardized using a z-transformation technique, giving all characters a mean of 0 and a variance of 1 (Anderson et al. 2011). A standardized matrix of biomechanical character scores was then subjected to principal coordinates analysis (PCoA), using the Gower model to correct for missing data to produce biomechanical morphospace plots. PCoA and creation of morphospace plots was performed in Past, Version 3 (Hammer et al. 2001).

**Table 1:** Continuous biomechanical characters used in this study.

Code	Functional trait	Description
C1	Anterior mechanical advantage	Ratio of maximum out-lever (on functional tooth row) and jaw muscle in-lever moment arms
C2	Posterior mechanical advantage	Ratio of minimum out-lever (on functional tooth row) and jaw muscle in-lever moment arms
C3	Opening mechanical advantage	Ratio of maximum out-lever and opening in-lever moment arms
C4	Maximum aspect ratio	Proxy for maximum flexural stiffness in the jaw
C5	Average aspect ratio	Proxy for average flexural stiffness across the entire jaw
C6	Relative adductor fossa length	Length of adductor muscle attachment; proxy for jaw muscle size
C7	Relative dental row length	Length of functional tooth row relative to total jaw length
C8	Relative articular offset	Proxy for deviation of biting action from scissor-like mastication.
C9	Relative mandibular fenestra	Area of mandibular fenestrae relative to total lateral jaw area
C10	Relative dental curvature	Curvature of functional tooth row; proxy for shearing vs. compressive mastication
C11	Cheek tooth height:breadth	Proxy for maximum tooth size for teeth occluding with maxillary teeth
C12	Premaxilliary occluding tooth height:breadth	Proxy for maximum tooth size for teeth occluding with premaxillary teeth
C13	Tooth packing	Proxy for tooth separation and how closely teeth are packed
C14	Predentary tooth procumbancy	Proxy for anterior-most tooth procumbancy
C15	Tooth height:jaw depth	Height of tooth present above deepest section of functional jaw taken
C16	Relative symphyseal length	Proxy for robustness of anterior jaw
C17	Mandibular symphysis orientation	Proxy for symphyseal resistance to bending during biting
C18	Predentary offset	Proxy for predentary curvature in ornithischians

Significant differences in morphospace occupation were tested using nonparametric multivariate analysis of variance (NPMANOVA) in Past, Version 3 (Hammer et al. 2001). All principal axes accounting for more than 1% of variation were used in the NPMANOVA, resulting in 12 axes for shape-based and 15 axes for biomechanical morphospace. Principal axes were used to display two types of morphospace comparisons: overall shape-based and biomechanical morphospace between sauropodomorphs and ornithischians. We also created a series of morphospace plots representing eight 20 Myr time slices. These time slices were constructed by combining taxa from two adjacent 10Myr time bins used for the disparity analyses (see following section). Combining time bins allowed for good sample size and enabled comparisons across major ecological transitions, for example, mass extinction events.

#### Disparity

Disparity through time was calculated across sixteen 10Myr time bins. The lengths of the time bins either side of the Tr/J boundary were adjusted to accommodate the date of the boundary as in Butler et al. (2012). Use of 10Myr time bins enables comparisons across both the Tr/J and J/K boundaries, standardizes bin length, and provides greater sample sizes per bin than those available for strict stage-level comparisons. Sauropodomorph disparity was also analyzed for vertical feeding envelopes in 3 m intervals. Species assignment to each maximum feeding envelope is listed in the Supplementary Material. To account for variation in the published literature, maximum sauropodomorph feeding envelopes were taken from published works, including reconstructions from new material (e.g., Upchurch and Barrett 2000; Apesteguía 2004; Sander et al. 2006; Peyer and Allain 2010; Whitlock 2011; Stevens 2013). Disparity analyses were carried out using the Morphological Disparity Analysis (MDA) package for Matlab (Navarro 2003). For all disparity tests, two variance-based disparity metrics were tested: the sum of variance and mean pairwise distance. Both these metrics are robust to sample size variation (Ciampaglio et al. 2009). The sum of variance metric is plotted in the main text. Mean pairwise distance results can be viewed in the Supplementary Material. Data were bootstrapped (1000 replicates), and 95% confidence intervals were calculated and graphically presented. Significant differences and likelihood ratios between each time bin were calculated using pairwise *t*-tests and marginal-likelihood assessment on sum of variance measures (Finarelli and Flynn 2007). A likelihood ratio >8 is considered a likely result (Finarelli and Flynn 2007). Results of *t*-tests were subsequently corrected for multiple comparisons, using Bonferroni corrections where appropriate (Holm 1979). Results for mean pairwise distance can be found in the Supplementary Material.

## Results

### 

#### Shape Morphospace Occupation

**Figure 1:**
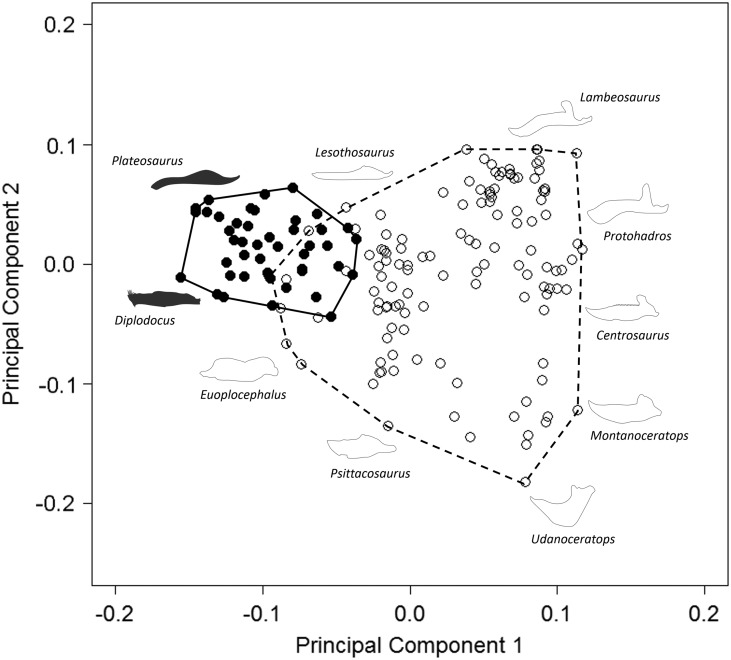
Patterns of morphospace occupation for herbivorous nonavian ornithischian and sauropodomorph dinosaurs. PC1 and PC2 account for 50.4% of variation. Ornithischian and sauropodomorph taxa occupy significantly different regions of shape-based morphospace (*p*<0.05). Filled circles, Sauropodomorpha; open circles, Ornithischia. Silhouettes represent jaw profiles found in that region of morphospace.

Our results demonstrate that sauropodomorph and ornithischian jaws occupy significantly different regions of morphological morphospace (*p*<0.01; Fig. 1; Table 2). There is minimal overlap between sauropodomorphs and ornithischians along PC1, with only seven ornithischian jaw morphologies occupying similar regions to sauropodomorphs. Overlapping ornithischian taxa represent basal members of their respective groups (basal ornithischians: *Agilisaurus* and *Pisanosaurus*; thyreophorans *Emausaurus* and *Gigantspinosaurus*; and the basal ceratopsian *Yinlong*), with the exception of *Stegosaurus* (two species). Regions of overlap are occupied by a wide range of both basal and derived sauropodomorphs; these include: *Plateosaurus gracilis*, *Lamplughsaura*, mamenchisaurids, brachiosaurids, and two South American titanosaurids (*Antarctosaurus* and *Bonitasaura*). Sauropodomorphs occupy morphospace exclusively in the −PC1 region: this region is characterized by dorsoventrally narrow jaws and the lack of a prominent coronoid process. Noneusauropod sauropodomorphs (e.g., *Plateosaurus*, *Melanorosaurus*), for the most part, account for sauropodomorph occupation of morphospace in +PC2: this region is typified by very narrow anterior jaws. Macronarian and diplodocoid taxa (including *Diplodocus* and *Tapuiasaurus*) primarily occupy −PC2 regions of morphospace (Fig. 1). The center of the morphospace (0.0 PC1; 0.0 PC2) is occupied by nonhadrosaurid iguanodontians (*Parksosaurus*, *Theiophytalia*, and *Dryosaurus*). Jaws in this region exhibit a greater gap between landmarks 1 and 2 than in sauropodomorph morphospace (due to the presence of the predentary in iguanodontians). Disparate groups of nonthyreophoran ornithischians expand morphospace occupation into +PC1 and +PC2 (hadrosaurids) and −PC2 regions (leptoceratopsids and psittacosaurids). +PC1 and +PC2 regions typically contain jaws with prominent coronoid processes and downwardly deflected predentaries; −PC2 regions contain robust, dorsoventrally broad jaws. Nonceratopsid marginocephalian jaw morphologies, such as those of psittacosaurids and leptoceratopsids, contribute strongly to the expansion of ornithischian shape morphospace, predominantly into +PC1/−PC2. Taxa are absent in a region of morphospace around +0.05 PC1/−0.075 PC2.

**Table 2:** Results of significance testing (NPMANOVA) on morphospace occupation (PC1 and PC2) and biomechanical occupation (PCo1 and PCo2; PCo1 and PCo3) between Ornithischia and Sauropodomorpha (at *p*<0.05).

Shape–based morphospace	Sauropodomorpha	Ornithischia
Sauropodomorpha	—	<0.001
Ornithischia	<0.001	—
Biomechanical morphospace	Sauropodomorpha	Ornithischia
Sauropodomorpha	—	<0.001
Ornithischia	<0.001	—

#### Biomechanical Morphospace Occupation

**Figure 2:**
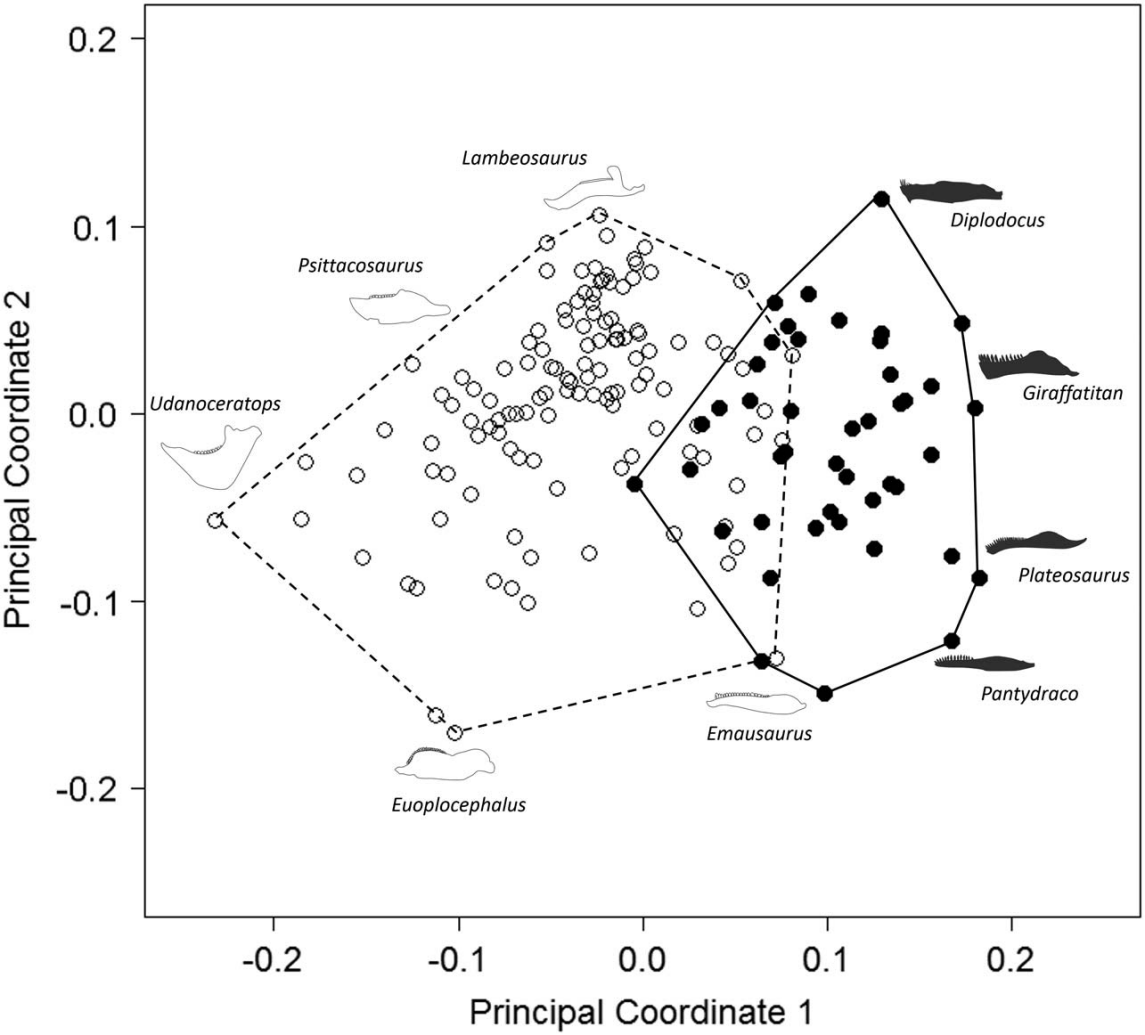
Patterns of biomechanical morphospace occupation for herbivorous nonavian ornithischian and sauropodomorph dinosaurs. PCo1 and PCo2 account for 25.2% of variation. Ornithischian and sauropodomorph taxa occupy significantly different regions of biomechanical morphospace (*p*<0.05). Filled circles, Sauropodomorpha; open circles, Ornithischia. Silhouettes represent jaw biomechanical profiles found in that region of biomechanical morphospace.

**Figure 3:**
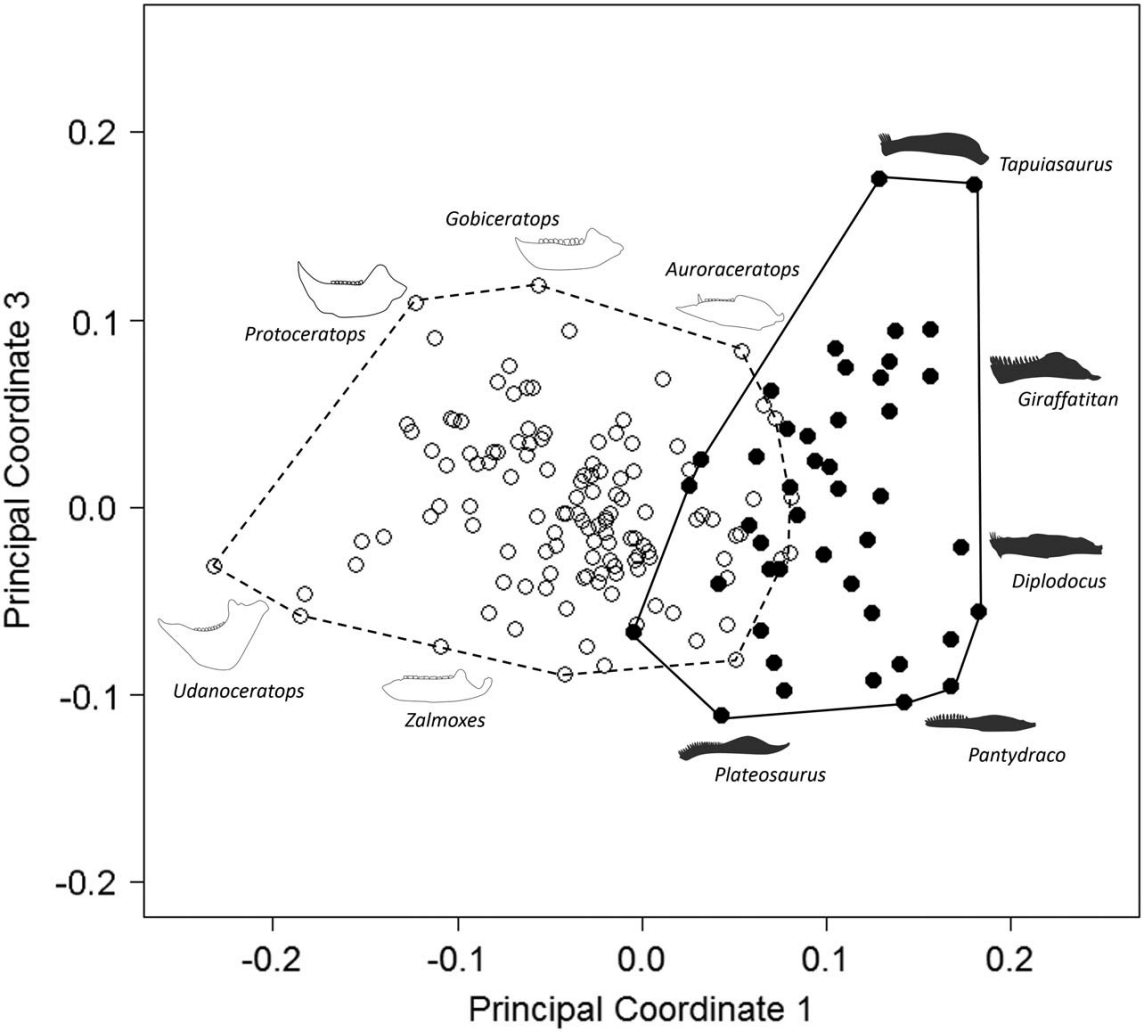
Patterns of biomechanical morphospace occupation for herbivorous nonavian ornithischian and sauropodomorph dinosaurs. PCo1 and PCo3 account for 23.9% of variation. Ornithischian and sauropodomorph taxa occupy significantly different regions of biomechanical morphospace (*p*<0.05). Filled circles, Sauropodomorpha; empty circles, Ornithischia. Silhouettes represent jaw biomechanical profiles found in that region of biomechanical morphospace.

Our results demonstrate that sauropodomorph and ornithischian taxa also occupy significantly different regions of biomechanical morphospace (*p*<0.01; Figs. 2, 3; Table 2). There is greater overlap in biomechanical morphospace occupation than shape morphospace, with 16–20 ornithischian taxa occupying morphospace that is shared with sauropodomorphs (Figs. 2, 3). Overlapping ornithischian taxa include basal ornithischians (*Pisanosaurus*, heterodontosaurids) and basal members of Thyreophora (*Emausaurus*, stegosaurs), Marginocephalia (*Yinlong*), and Ornithopoda (*Changchunsaurus*, *Dysalotosaurus*). Sauropodomorphs occupy regions of +PCo1. Noneusauropod sauropodomorphs (e.g., *Coloradisaurus*, *Pantydraco*) predominate in +PCo1/−PCo2. This region is characterized by jaws with a high mechanical advantage and a large adductor muscle attachment area. Diplodocids, nonneosauropods, and nontitanosaurian macronarians (e.g., *Mamenchisaurus*, *Camarasaurus*) stretch sauropodomorph occupation into +PCo2. Jaws in this region also display high mechanical advantages, coupled with high aspect ratios. Many iguanodontian, ceratopsid, and psittacosaurid jaw profiles occupy similar regions of +PCo2 biomechanical morphospace (Fig. 2). Occupation is spread deeper into −PCo1 by leptoceratopsids (e.g., *Montanoceratops*). This region of functional space is characterized by deep jaws with short adductor muscle attachment and a high posterior mechanical advantage. Expansion into −PCo2 is accounted for by deep-jawed ankylosaurs (*Euoplocephalus*, *Silvisaurus*), with low tooth:jaw depth ratios and high relative dental length (Fig. 2). Similar patterns are observed in PCo3, with more basal sauropodomorphs occupying −PCo3, with a large cluster of iguanodontians and ceratopsids occupying regions of central morphospace (0.0 PCo1; 0.0 PCo3). Functional loadings, interpretations for the first four principal axes, and individual species placement in morphospace can be found in the Supplementary Material.

#### Morphospace Occupation through Time

**Figure 4:**
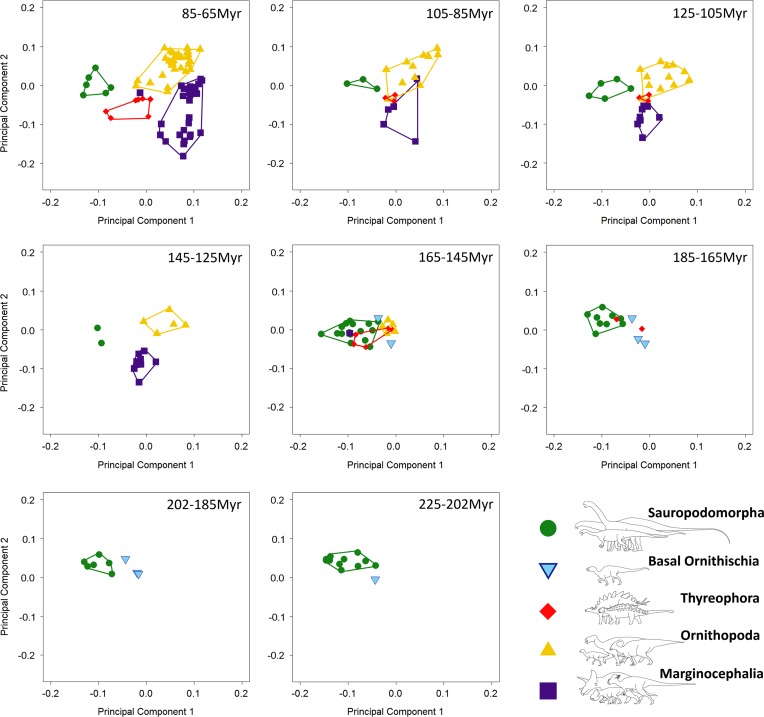
Patterns of morphospace occupation for herbivorous nonavian dinosaurs through the Mesozoic (20 Myr time bins), based on PC1 and PC2 (accounting for 50.4% of variation). Sauropodomorpha occupy isolated regions of morphospace for the majority of the Mesozoic, with overlap between North American sauropods and thyreophorans between 185 and 145 Ma.

**Figure 5:**
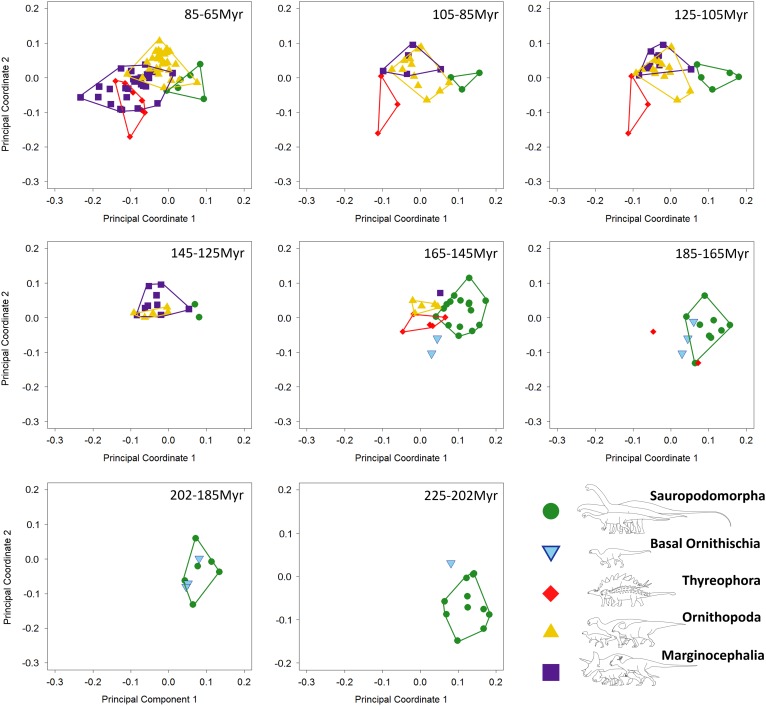
Patterns of biomechanical morphospace occupation for herbivorous nonavian dinosaurs through the Mesozoic (20 Myr time bins), based on PCo1 and PCo2 (accounting for 25.2% of variation). Sauropodomorphs predominantly overlap only with heterodontosaurids (202–145 Ma). Aptian–Maastrichtian marginocephalians and ornithopods occupy similar regions of morphospace (125–65 Ma).

**Figure 6:**
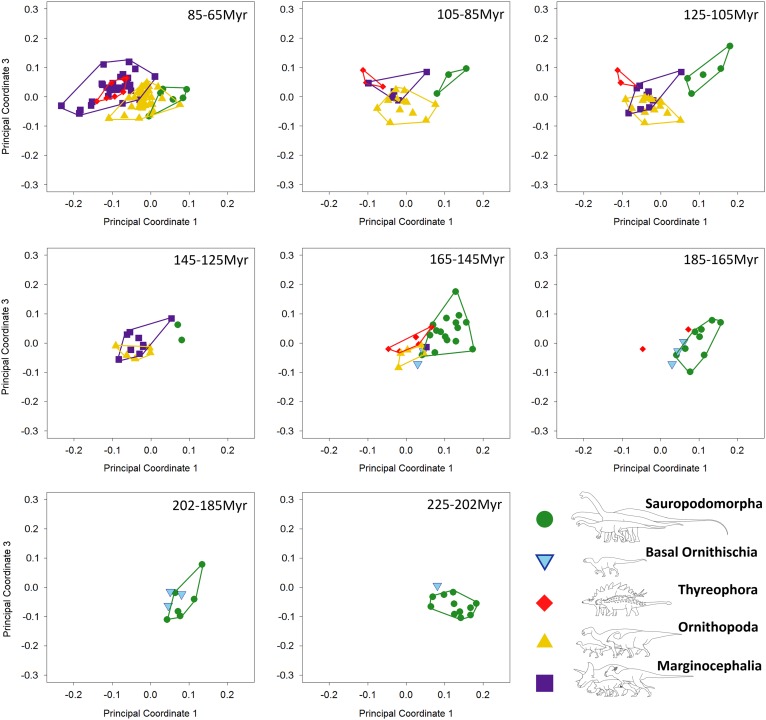
Patterns of biomechanical morphospace occupation for herbivorous nonavian dinosaurs through the Mesozoic (20 Myr time bins), based on PCo1 and PCo3 (accounting for 23.9% of variation). Sauropodomorphs overlap very little with contemporaneous taxa before the latest Cretaceous (85–65 Ma). Albian–Maastrichtian marginocephalians and thyreophorans occupy similar regions of biomechanical morphospace (105–65 Ma).

Breakdown of shape and biomechanical morphospace into 20 Myr time bins highlights patterns of morphospace occupation by each clade through time (Figs. 4–6). Initial occupation during the Late Triassic–Middle Jurassic is dominated by sauropodomorphs, with low numbers of contemporaneous basal ornithischians (e.g., heterodontosaurids and thyreophorans). In the bin representing the 20 Myr prior to the J/K boundary (165–145 Ma), thyreophorans, ornithopods, marginocephalians, and heterodontosaurids all occupy similar regions of shape morphospace, yet at this time, the same clades occupy disparate regions of biomechanical morphospace with little overlap (Figs. 5, 6; 165–145 Ma, Table 3). Sauropodomorphs at this time show significantly different biomechanical occupation to stegosaurs and ornithopods, but not heterodontosaurids or the basal ceratopsian *Yinlong* (NPMANOVA, *p*<0.01; Table 3). The sauropodomorphs are biomechanically diverse prior to the J/K boundary, occupying the region of morphospace that correlates to high tooth height:base, high mechanical advantages, and large mandibular fenestrae. After the J/K boundary, morphospace and biomechanical morphospace plots show a drop in sauropodomorph morphological and biomechanical variation as sample size diminishes and expansion in disparity by marginocephalians and, later, ornithopods (Figs. 4–6, 145–65 Ma). By the Early Cretaceous, the surviving Jurassic herbivorous dinosaur clades (sauropodomorphs, marginocephalians, ornithopods, and thyreophorans) are statistically distinct in both shape and biomechanical morphospace (Table 2). Sauropodomorphs display substantially reduced variation, whereas ankylosaurs, ceratopsians, and ornithopods expand into hitherto unoccupied regions of biomechanical morphospace. Marginocephalians (e.g., *Psittacosaurus*) share areas of biomechanical morphospace with iguanodontians but occupy very different regions of shape space (Fig. 4, 145–105 Ma).

**Table 3:** NPMANOVA significance testing between clade occupations of biomechanical morphospace through time. Bold *p*-values represent significant differences (at *p*<0.05). SA, Sauropodomorpha; BO, Basal Ornithischia; TH, Thyreophora; OR, Ornithopoda; MA, Marginocephalia.

Time bin	NPMANOVA *p*-values					
	Clades	SA	BO			
225–202 Ma	SA	—	0.114			
	BO	0.114	—			
	Clades	SA	BO			
202–185 Ma	SA	—	**0.009**			
	BO	**0.009**	—			
	Clades	SA	BO	TH		
185–165 Ma	SA	—	0.142	1		
	BO	0.142	—	1		
	TH	1	1	—		
	Clades	SA	BO	TH	OR	MA
165–145 Ma	SA	—	0.505	**0.009**	**0.015**	1
	BO	0.505	—	0.520	0.124	1
	TH	**0.009**	0.520	—	0.158	1
	OR	**0.015**	0.124	0.158	—	1
	Clades	SA	OR	MA		
145–125 Ma	SA	—	0.084	**0.003**		
	OR	0.084	—	**0.016**		
	MA	**0.003**	**0.016**	—		
	Clades	SA	TH	OR	MA	
125–105 Ma	SA	—	0.186	**<0.001**	**<0.001**	
	TH	0.186	—	**0.003**	**0.007**	
	OR	**<0.001**	**0.003**	—	**<0.001**	
	MA	**<0.001**	**0.007**	**<0.001**	—	
	Clades	SA	TH	OR	MA	
105–85 Ma	SA	—	0.164	**0.002**	**0.043**	
	TH	0.164	—	**0.005**	**0.037**	
	OR	**0.002**	**0.005**	—	**<0.001**	
	MA	**0.043**	**0.037**	**<0.001**	—	
	Clades	SA	TH	OR	MA	
85–65 Ma	SA	—	**<0.001**	**<0.001**	**<0.001**	
	TH	**<0.001**	—	**<0.001**	**<0.001**	
	OR	**<0.001**	**<0.001**	—	**<0.001**	
	MA	**<0.001**	**<0.001**	**<0.001**	—	

In the latest Cretaceous, the four clades present occupy distinct regions of shape morphospace (*p*<0.01; Table 2), with the exception of one marginocephalian taxon (*Stegoceras*) that plots between nonhadrosaurid ornithopods and ankylosaurians (Fig. 4, 85–65 Ma). Biomechanically, *Stegoceras* is nested among ornithopods and is closer to sauropods than many contemporaneous ceratopsians. Corresponding biomechanical morphospace plots show a very different trend. Marginocephalians overlap with both ornithopods and thyreophorans. Thyreophorans and ornithopods do not overlap, and sauropodomorphs overlap minimally with ornithopods (Figs. 5, 6, 85–65 Ma). Whereas variation in marginocephalian jaw shape and biomechanics increases throughout the Cretaceous, ornithopod shape and biomechanical variation remains constant throughout the Late Cretaceous. Leptoceratopsids (e.g., *Udanoceratops*, *Montanoceratops*) extend biomechanical morphospace occupation into the region of morphospace characterized by deep mandibles with short adductor muscle attachment and high posterior mechanical advantages (Figs. 5, 6). Full details of the biomechanical character loadings are described in the Supplementary Appendix 5.

#### Disparity

**Figure 7:**
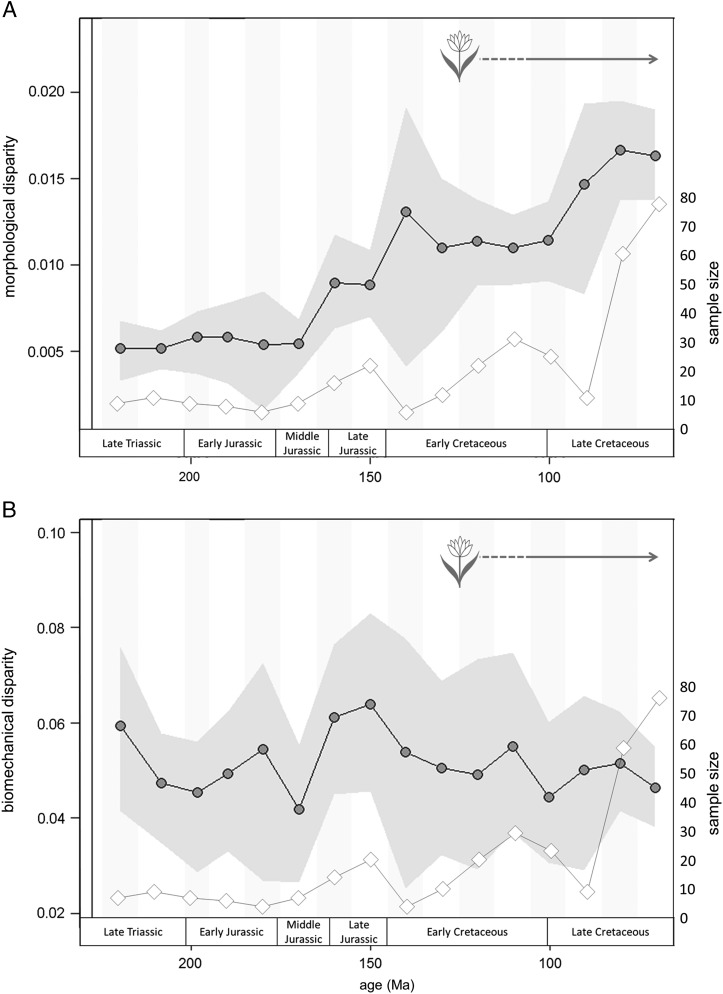
Comparison of shape-based and biomechanical disparity curves across 10 Myr time bins based on sum of variance metric. (A) shape-based disparity; (B) biomechanical disparity. Morphological and biomechanical disparity curves are decoupled, with morphological disparity increasing through the Mesozoic and biomechanical disparity peaking in the latest Jurassic. Shaded region spans the 95% confidence intervals based on 1000 bootstrap replicates. Disparity (dots) is plotted alongside jaw specimen sample size curve (diamonds). Flower represents earliest fossil angiosperms (Sun et al. 2002; Du and Wang 2015).

Morphological (shape) and biomechanical disparity measures are decoupled through the Mesozoic (Fig. 7). Morphological disparity primarily tracks sample diversity (Fig. 7A): it does not fluctuate greatly through the first 80 Myr of dinosaur evolution, begins to increase from the Middle Jurassic onward, and reaches a peak in the Late Cretaceous (Fig. 7A). There are no significant differences in disparity between time bins (*p*>0.05). By contrast, biomechanical disparity undulates through the Mesozoic (Fig. 7B), a decoupling from sample diversity and morphological diversity. Several small peaks and troughs (for example the peak in the Late Jurassic) correspond to increased sample size (Fig. 7B, diamond data points): however, time periods with greatest sample sizes do not correspond to peaks in biomechanical disparity (during the latest Cretaceous, for example). The peak in the latest Jurassic also corresponds with the presence of high-browsing sauropodomorphs (>9 m), which display a higher degree of biomechanical disparity than some lower-browsing forms (*p*>0.05; see Supplementary Fig. 10). There are no significant differences in disparity between successive time bins for either biomechanical or morphological disparity curves (at *p*=0.05) and no marginal-likelihood values exceed the threshold value of 8. There are a few instances where disparity diverges markedly from sample size, suggesting that a trend, albeit nonsignificant, might be observed. For example, morphological disparity rises in the Early Cretaceous, immediately after the J/K extinction, and in the early Late Cretaceous, while sample size drops. Likewise, biomechanical disparity drops in the Middle Jurassic while sample size rises slightly. Conversely, in the latest Cretaceous, sample size rises sharply while biomechanical disparity drops very slightly.

## Discussion

### 

#### Impact of Extinction on Herbivorous Dinosaur Disparity

Our results from both morphological and biomechanical disparity curves support conclusions from previous studies examining dinosaur disparity around extinction events (Brusatte et al. 2008a, 2012). Morphological disparity across the Tr/J boundary increases slightly, likely triggered by the addition of heterodontosaurid jaw profiles to the morphospace (Fig. 7B). Biomechanical disparity decreases from an initial peak in the Carnian (225 Ma) to the Tr/J boundary, across which there is a further nonsignificant decrease (Fig. 7B). The placement of taxa in biomechanical morphospace suggests that both ornithischian and sauropodomorph taxa share similar biomechanical profiles immediately before and after the Tr/J boundary (Figs. 5, 6). By contrast, the transition across the J/K boundary shows a decoupled relationship between biomechanical and morphological disparity (Fig. 7). Morphological disparity after the J/K boundary increases sharply: this pattern can be attributed to the presence of novel jaw morphologies such as those of psittacosaurids and early hadrosauroids in combination with those of new sauropod clades (Fig. 4, 145–125Ma). It should be noted that this disparity increase is nonsignificant, likely due to the low taxon count (*n*=5). The lack of many dinosaur-bearing formations between the Berriasian and Albian may partially account for the low species richness observed in this interval, although it could also be attributed to the J/K extinction event (Barrett et al. 2009; Upchurch et al. 2011). Nevertheless, shape variation at this time does not track sample diversity. Biomechanical disparity shows a decrease across the J/K boundary (Fig. 7B). The majority of the biomechanical profiles exhibited prior to the J/K boundary do not persist into the earliest Cretaceous (Figs. 5, 6, 145–125Ma), which is consistent with the fundamental faunal turnover that takes place and the proliferation of marginocephalian and ornithopod taxa (e.g., Bakker 1978; Weishampel and Norman 1989; Barrett and Willis 2001). Finally, our results concur with disparity patterns observed in the latest Cretaceous leading to the Cretaceous/Paleogene (K/Pg) mass extinction (Brusatte et al. 2012): both morphological and biomechanical curves show a decrease in disparity from the Campanian to the Maastrichtian, despite a notable increase in sample size.

#### Patterns of Morphospace Occupation

Discrete morphospace occupation suggests that, when considered as a single data set, the jaws of sauropodomorphs and ornithischians are different in both shape and in jaw biomechanics (Figs. 1–3). Individual occupation of morphospace by each taxon is graphically represented in Supplementary Figures 2–6. Limited overlap between these clades suggests little competition between ornithischians and sauropodomorphs in feeding function, particularly during the latter part of the Mesozoic (see also Barrett and Upchurch 2005). However, where overlap does occur, it tends to be between the basal members of various ornithischian clades (e.g., heterodontosaurids, basal thyreophorans, and basal ceratopsians) and sauropodomorphs. This suggests that early ornithischians adopted similar morphological and mechanical attributes to their feeding apparatus as macronarian sauropodomorphs (Supplementary Fig. 2, a–c). Later groups of ornithischians radiated into distinct areas of morphospace (Figs. 4–6). Breakdown of morphological and biomechanical morphospace into 20 Myr time bins shows that earlier sauropodomorphs are, in general, replaced in their biomechanical profiles by later sauropodomorphs through the Jurassic and Cretaceous (Figs. 4–6). Sauropodomorph morphospace occupation shows a degree of migration through time, with basal sauropodomorphs occupying different regions of morphospace to Jurassic and Cretaceous neosauropods (Figs. 4–6, filled circles). Some later sauropods show convergence in biomechanical profile with other, earlier forms. For example, the macronarian *Camarasaurus* occupies very similar regions of morphospace to the earlier diverging eusauropod *Datousaurus* (Supplementary Fig. 2a–c), despite the former existing around 10Myr earlier: this pattern supports the results of another recent quantitative craniodental study (Button et al. 2014). Similarly, the titanosaurid *Antarctosaurus* occupies biomechanical morphospace almost identical to the basal macronarian *Abrosaurus* (Supplementary Fig. 2a–c). Perhaps surprisingly, we find minimal convergent occupation in biomechanical morphospace between titanosaurids (e.g., *Antarctosaurus*) and diplodocids (e.g., *Diplodocus*) (Supplementary Fig. 2a–c: see also Button et al. 2014). This pattern is in contrast to shape-based morphospace (this study), in which these groups occupy similar regions of morphospace (Fig. 1 and Supplementary Fig. 2). Both shape-based and biomechanical morphospace patterns show extensive overlap between phylogenetically separate groups of sauropodomorphs. Within the sauropods, brachiosaurids are found to be biomechanically intermediate between basal macronarian sauropods with short snouts and closely packed tooth rows (such as *Camarasaurus*) and titanosaurids with longer snouts and pencil-like teeth (such as *Antarctosaurus*), and diplodocids are outliers in this biomechanical morphospace. This pattern supports quantitative work on sauropodomorph cranial morphology related to feeding, with similar placement of the same taxa in cranial (Button et al. 2014) and mandibular morphospace (this study). Late Jurassic sauropods such as *Camarasaurus* show some morphological overlap in mandibular shape with stegosaurs. By contrast, these same clades show minimal overlap in biomechanical morphospace: only *Gigantspinosaurus* (Stegosauria) and *Manidens* (Heterodontosauridae) share occupation of Late Jurassic sauropodomorph biomechanical morphospace (Supplementary Figs. 3,b–c, 4,b–c). This suggests that mandibles with similar gross morphology were biomechanically and functionally differentiated by this time. In general, sauropodomorphs and heterodontosaurids occupy similar regions of both shape-based and biomechanical morphospace and do not extend their occupation of morphospace beyond regions already occupied by the end of the Early Jurassic (Figs. 4–6). From the Middle Jurassic onward, there is slight expansion of morphospace along PC1 by diplodocoid sauropodomorphs and Jurassic ornithopods (e.g., *Camptosaurus*), which is also reflected in the morphological disparity curve (Fig. 4, 165–145 Ma; Fig. 7A). Morphological disparity shows an increase from the latest Jurassic through the Cretaceous with the evolution of new groups of ornithischian dinosaurs, particularly marginocephalians.

Early Cretaceous marginocephalians (psittacosaurids, *Archaeoceratops*, and *Liaoceratops*) occupy novel regions of morphological and biomechanical morphospace: these taxa share regions of biomechanical morphospace with hadrosauroids until the disappearance of basal marginocephalians prior to the last 20 Myr of the Mesozoic (Figs. 4–6, 85–65 Ma). Regions of biomechanical morphospace formerly occupied by psittacosaurids were then occupied exclusively by derived hadrosaurids and ankylosaurs (Figs. 5, 6, 85–65 Ma). However, the morphological profile of psittacosaurids was never replaced. The latest Cretaceous sees an expansion of biomechanical and shape-based morphospace by two distinct groups of marginocephalians: ceratopsids (e.g., *Triceratops*) and leptoceratopsids (e.g., *Udanoceratops*). The biomechanical profiles of ceratopsids show no overlap with those of hadrosaurids. This supports the conclusions of Mallon and Anderson (2013) who, in their study of herbivores from the Dinosaur Park Formation (Campanian), found that contemporaneous hadrosaurids, ankylosaurs, and ceratopsids occupied different feeding niches based upon differing cranial and mandibular mechanics and morphologies. This study also supports previous conclusions on niche partitioning between hadrosaurs and ceratopsids (Mallon and Anderson 2013). However, this study also found that the majority of derived ceratopsids plot in similar regions of biomechanical morphospace to contemporaneous ankylosaurs, in contrast to the conclusions of Mallon and Anderson (2013). In addition, Asian ankylosaurs show biomechanical morphospace occupation more similar to leptoceratopsids than to ceratopsids or North American ankylosaurs. It should be noted, however, that neither leptoceratopsids nor Asian ankylosaurs were included in Mallon and Anderson (2013), which focused solely on the Dinosaur Park Formation fauna. Leptoceratopsids expand into regions of shape-based and biomechanical morphospace that had no previous occupants: their extreme mandibular morphologies account for the peak in morphological disparity in the latest Cretaceous (Fig. 7A). Contemporaneous taxa include ceratopsids and ankylosaurs that have similar biomechanical profiles to each other (see above). This biomechanical similarity would cause disparity to be low: however, the inclusion of the highly disparate leptoceratopsids (in addition to hadrosaurids and the rhabdodontid *Zalmoxes*) leads to an increase in biomechanical disparity levels from the early Late Cretaceous. Marginocephalian, ornithopod, and thyreophoran biomechanical morphospace occupation in the latest Cretaceous suggests that these groups, while varying from each other in mandibular shape, also share a variety of functional and biomechanical traits relating to feeding. Late Cretaceous hadrosaurids and ankylosaurids filled the biomechanical roles vacated by Early Cretaceous nonhadrosaurid iguanodontians and nodosaurids, respectively. Individual occupation of morphospace by each taxon can be viewed in Supplementary Figures 2–6.

#### Dinosaur–Plant Coevolution

Changes in dinosaur communities and feeding regimes during the Late Jurassic–Early Cretaceous interval have been linked to several major floristic changes (decline of cycadophytes, gymnosperms, and pteridophytes; rise of angiosperms to ecological dominance) (e.g., Weishampel and Norman 1989; Tiffney 1992; Mustoe 2007). Our results provide quantitative evidence that the mandibles of sauropodomorphs and ornithischians evolved different morphologies and biomechanical profiles, potentially enabling them to feed on different plants in different ways. Moreover, their minimal overlap in biomechanical morphospace suggests that there was limited competition between ornithischians and sauropodomorphs when feeding (see also Barrett and Upchurch 2005). Our data demonstrate that there was no significant increase in the biomechanical disparity of the feeding apparatus of either major herbivorous dinosaur clade that was coincident with the proliferation of angiosperms (Fig. 7). Nevertheless, although this novel food source appears to have had no discernible impact on the mandibular biomechanical morphospace occupation of herbivorous dinosaurs, patterns of morphological disparity do show a marked increase coincident with the later Cretaceous proliferation of angiosperms. This coincident increase is not interpreted as indication of direct causality, but reflects the appearance of the highly disparate ankylosaurid and leptoceratopsian jaw morphotypes.

Potential links to cycadophyte decline through the Late Jurassic–Early Cretaceous are less clear. The Early Cretaceous decline in cycadophytes occurred at a time of major faunal change affecting dinosaur clades, but previous analyses of dinosaur and plant distribution have shown that few of the observed changes in dinosaur faunas could be linked directly with cycadophyte decline (Butler et al. 2009b). Although reduced biomechanical mandibular disparity across the J/K boundary does coincide with the onset of this event, direct evidence of dinosaur herbivory on cycads is sparse (Hummel et al. 2008; Butler et al. 2009b; Gee 2011), and other causes relating to the poorly understood J/K extinction may also be involved (Butler et al. 2011; Upchurch et al. 2011). In addition, morphological disparity after this extinction event shows a notable increase, with different clades of dinosaurs diversifying into new, unexplored regions of mandibular morphospace (e.g., psittacosaurids, early titanosaurs). Results from this study do not support a coevolutionary relationship between herbivorous dinosaur mandibular disparity and angiosperm proliferation and show a similarly negative relationship to the decline of cycadophytes. Rather, patterns of mandibular shape and mechanical diversity seem to be most greatly affected by the extinction and emergence of different dinosaurian clades.

#### Sampling Issues

When disparity tracks sample diversity closely, as it does in this study for shape-based disparity, sampling bias cannot be ruled out. Morphological disparity in this study partly tracks jaw sample size, suggesting a potential bias in the data set for some features of the disparity curve (e.g., high sample and disparity in latest Cretaceous; Fig. 7A). The use of the sum of variance disparity measure and bootstrapping the data has accounted for sample size as best as is possible for the data set (Foote 1992, 1994; Ciampaglio et al. 2009) (Fig. 7A). Peaks of high shape disparity in the earliest Cretaceous and early Late Cretaceous do not correlate with peaks in sample size. Biomechanical variation displays a different trend, demonstrating a decoupling of morphological and biomechanical diversity through time. A peak in biomechanical disparity in the Late Jurassic is coincident with an increase in jaw sample size, but also corresponds to the evolution of high-browsing (>9 m) sauropods (e.g., Upchurch and Barrett 2000). In addition, many of the sauropod taxa in this time slice are recovered from the Morrison Formation of the western United States (*n*=6 out of a total of 14 sauropods). The exclusion of the Morrison taxa removes the Late Jurassic peak in biomechanical disparity (Supplementary Fig. 8i). A similar jackknifing of the taxa from the Dashanpu Formation (including the “Upper and Lower Shaximiao” formations) yielded a trough in disparity in the Middle Jurassic but retained a strong peak in the latest Jurassic (Supplementary Fig. 8ii). These results suggest that the data may be sensitive to the inclusion or exclusion of particularly rich fossil-bearing sites. In addition, the lack of available jaw material from North and South American titanosaurs seriously underrepresents sauropodomorph diversity in the Cretaceous. The addition of titanosaurid taxa to the analysis may increase both the disparity and overall morphospace occupation of sauropodomorphs, although the titanosaur jaws sampled in this study already account for a broad range of morphologies (Supplementary Fig. a–c, taxon 37–44).

Supplementary analyses of biomechanical and shape-based disparity within sauropodomorphs in relation to maximum feeding height show higher levels of disparity in high-browsing sauropods (>9 m; e.g., *Brachiosaurus*, *Mamenchisaurus*) when compared with mid-browsing taxa (6–9 m; e.g., *Camarasaurus*), and almost equal in disparity to very low-browsing sauropodomorphs (0–3 m; e.g., *Pantydraco*, *Riojasaurus*) (see Supplementary Fig. 10). This pattern contrasts with sample diversity, with the lowest sample size found in the high-browsing feeding envelope (*n*=6) (Supplementary Fig. 10). Unfortunately, low sample sizes within each feeding level prevent any significant differences or definitive conclusions to be made. However, this pattern remains intriguing and the addition of more mandibular remains from high- and mid-browsing taxa to our sample (as and when they are discovered) would complement this study. This is an avenue of study that requires more investigation in the future to enable deeper insights into niche partitioning between sauropod groups based on maximum browse height.

Relatively few Early Cretaceous sauropodomorph, thyreophoran, or marginocephalian taxa possess well-preserved mandibular material (see list of taxa in Supplementary Material). The dip in biomechanical disparity after the J/K recovered by our analyses may, therefore, be an artefact due to either geological biases or uneven collection effort, underrepresenting the true diversity of jaw biomechanical profiles at this time. Due to the lack of complete mandibles from rebbachisaurids, dicraeosaurids, and other clades, it is possible that the latest Jurassic and earliest Cretaceous disparity levels reported herein are currently undersampling the total diversity of mandible morphology and potential function. Such exclusions cannot be corrected for by our analyses and represent a limitation of the fossil material currently available.

## Conclusions

For the first time, we have quantified the morphological and biomechanical variation of ornithischian and sauropodomorph jaws throughout the Mesozoic and examined how diversity related to external extrinsic drivers such as extinction events and the rise of angiosperms. We find that herbivorous dinosaur clades have jaws that occupy different regions of morphospace throughout the Mesozoic. Furthermore, sauropodomorphs and ornithischians have jaws that also function in broadly different ways, yet there is some potentially convergent overlap in biomechanical function between different ornithischian clades in the Cretaceous. Basal members of each clade tend to be more similar in form and function to each other, while derived taxa are more functionally and morphologically divergent. Herbivorous dinosaur jaws maintained a numerically steady diversity of biomechanical traits, with a peak observed in the Late Jurassic triggered by the diversification of high-browsing sauropods. This is consistent with a rapid evolutionary radiation in biomechanical diversity among herbivorous dinosaurs followed by a plateau. The Tr/J extinction had no overall effect on biomechanical variation among herbivorous dinosaurs, despite fundamental changes in floral and faunal composition across the boundary. This consistency suggests that Early Jurassic dinosaurs filled the functional feeding niches vacated by the extinction of Late Triassic taxa. Similar successive replacement patterns are also seen in Devonian gnathostomes and Devonian to mid-Pennsylvanian tetrapodomorphs (Anderson et al. 2011, 2013). Biomechanical disparity across the J/K boundary suggests that large-scale faunal turnover at this time did affect mandibular disparity, which did not recover to pre-J/K disparity levels through the Cretaceous (Fig. 7). A diverse fauna of high-browsing sauropods did not persist into the Early Cretaceous, and the sauropodomorph contribution to overall disparity wanes through the Cretaceous, despite a later increase in their Late Cretaceous species richness. The highly specialized psittacosaurids were not replaced in their biomechanical profile. However, their role as a biomechanically disparate group in Asia is later filled by Late Cretaceous leptoceratopsids (e.g., *Udanoceratops*), a group that is also present in North America. Late Cretaceous hadrosaurids and ankylosaurids filled the biomechanical roles vacated by Early Cretaceous nonhadrosaurid iguanodontians and nodosaurids respectively. Our results imply that, after the establishment of peak overall biomechanical variation in the latest Jurassic, only marginocephalians demonstrated widespread variation in biomechanical profiles over time, triggered by the isolated adaptive radiations of psittacosaurids and leptoceratopsians. The remainder of Cretaceous herbivorous dinosaurs underwent progressive niche replacement, with successive replacement by related taxa with comparable biomechanical profiles.

## Supplementary Material

*Data available from the Dryad Digital Repository*: http://dx.doi.org/10.5061/dryad.c78k5
